# Message From the New Editor‐In‐Chief: Advancing *AGSurg* Into a Leading International Surgical Journal

**DOI:** 10.1002/ags3.70151

**Published:** 2026-01-01

**Authors:** Ken Shirabe

**Affiliations:** ^1^ Division of Hepatobiliary and Pancreatic Surgery, Department of General Surgical Science, Graduate School of Medicine Gunma University Maebashi Japan

## Abstract

Trends in the Impact Factor of *Annals of Gastroenterological Surgery (Ag Surg)*. *AGSurg* achieved an impressive first impact factor of 5.164 in 2021. Although the impact factor temporarily declined to 2.7, it has since recovered, reaching 3.3 last year.

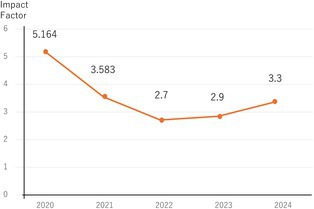

As President of the Japanese Society of Gastroenterological Surgery (JSGS) and the third Editor‐in‐Chief of *Annals of Gastroenterological Surgery (AGSurg)*, I would like to extend my heartfelt greetings to all our readers.

First, I wish to express my sincere gratitude and respect to everyone who has supported this journal to date—especially Dr. Masaki Mori, our founding Editor‐in‐Chief, who played a pivotal role in launching *AGSurg* and continues to guide us as Honorary Editor‐in‐Chief, and Dr. Yuko Kitagawa, our second Editor‐in‐Chief, who greatly contributed to the journal's development.

*AGSurg* was established with the vision that creating an official English‐language journal would enhance the international standing of our society and allow us to share our scientific insights more broadly with the global community. By raising international recognition of the high standard of gastroenterological surgery in Japan, we also aim to support the career development of Japanese surgeons. As an open‐access journal available worldwide, *AGSurg* plays a vital role in the internationalization of JSGS. Although the importance of internationalization has long been emphasized within academic societies, it seems that in recent years many young gastroenterological surgeons have remained focused mainly on domestic matters. For this reason, the role *AGSurg* should play in promoting internationalization is becoming even more significant.

The foundation for *AGSurg* was laid during Dr. Masaki Mori's presidency, and the journal was officially launched in 2017 under the leadership of Dr. Yasuyuki Seto. In its early stages, we invited leading experts in each field to contribute review articles, and many JSGS officers and members actively submitted high quality manuscripts. As a result, submissions increased rapidly, and numerous excellent original articles were published.

Thanks to this strong growth, *AGSurg* achieved an impressive first impact factor of 5.164 in 2021, just 4 years after its launch (based on 2020 data). Although the impact factor temporarily declined to 2.7, it has since recovered, reaching 3.3 last year. The journal's total citations have also steadily increased—from 864 in 2020 to 2062 in 2024 (Figure 1). We believe this progress reflects the passion and dedication of all who have supported *AGSurg*.

**FIGURE 1:**
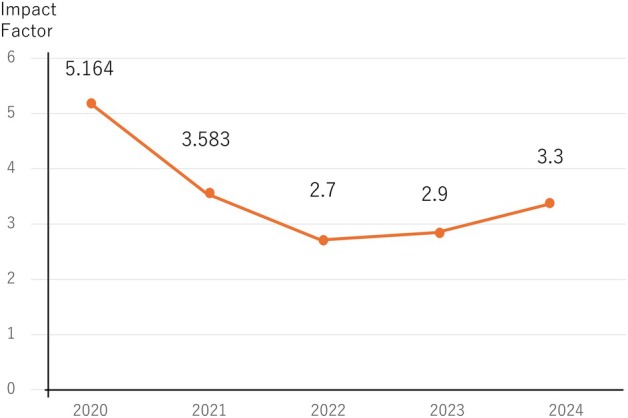
*AGSurg* achieved an impressive first impact factor of 5.164 in 2021. Although the impact factor temporarily declined to 2.7, it has since recovered, reaching 3.3 last year. The journal's total citations have also steadily increased—from 864 in 2020 to 2062 in 2024.

Looking ahead, our editorial team is united in its efforts to elevate *AGSurg* into one of the top journals in the field of surgery. Under the leadership of Dr. Koshi Mimori, Chair of the English Journal Committee, we are pursuing new initiatives such as publishing interviews with leading researchers and translating Japanese‐language clinical guidelines from other societies into English. Even so, we believe our most important mission is to remain committed to a traditional and rigorous approach: publishing high‐quality original research grounded in solid evidence. We firmly believe that the impact of a journal should ultimately reflect the impact of the individual articles it publishes.

Until now, *AGSurg* has adopted a deliberate and focused strategy to achieve rapid growth—soliciting submissions primarily from leading institutions in Japan and drawing on the networks of JSGS members. To become a truly international surgical journal, however, we recognize that our efforts toward internationalization must now enter a new phase. It is time for *AGSurg* to move beyond being a journal centered on Japanese members and to attract outstanding manuscripts from around the world. We therefore plan to welcome more young international researchers to our editorial and associate editorial boards and to actively seek high‐quality submissions from overseas, with the aim of transforming *AGSurg* into a truly global journal.

For many gastrointestinal surgeons, surgical treatment of gastrointestinal cancers is a crucial field. In recent years, the wave of minimally invasive surgery, such as endoscopic surgery and robot‐assisted surgery, has garnered significant interest among gastrointestinal surgeons. Numerous papers on these topics have been accepted in *AGSurg*. On the other hand, there is growing concern that the next generation of gastrointestinal surgeons may be losing interest in surgical oncology. While improving short‐term postoperative outcomes is undoubtedly an important issue for gastrointestinal surgeons, we must not forget that the ultimate goal of surgical treatment for gastrointestinal cancer is to improve long‐term oncological outcomes. In addition to papers focusing on surgical techniques, we would like to consider publishing content on surgical oncology in *AGSurg* with a balanced approach.

To all our readers, authors, and stakeholders, I sincerely ask for your continued support and cooperation. Through the ongoing development of *AGSurg*, we will continue striving to contribute to the advancement of gastroenterological surgery worldwide.

## Funding

The author has nothing to report.

## Conflicts of Interest

Dr. Ken Shirabe is a current member of the Editorial Board of AGS. The other author declares no conflicts of interest.

